# Identification of *Candida haemulonii* Complex Species: Use of ClinProTools^TM^ to Overcome Limitations of the Bruker Biotyper^TM^, VITEK MS^TM^ IVD, and VITEK MS^TM^ RUO Databases

**DOI:** 10.3389/fmicb.2016.00940

**Published:** 2016-06-16

**Authors:** Rafaella C. Grenfell, Afonso R. da Silva Junior, Gilda M. B. Del Negro, Regina B. Munhoz, Viviane M. F. Gimenes, Diego M. Assis, Anna C. Rockstroh, Adriana L. Motta, Flavia Rossi, Luiz Juliano, Gil Benard, João N. de Almeida Júnior

**Affiliations:** ^1^Department of Biophysics, Escola Paulista de Medicina, Universidade Federal de São PauloSão Paulo, Brazil; ^2^Central Laboratory Division – LIM-03, Hospital das Clínicas da Faculdade de Medicina, Universidade de São PauloSão Paulo, Brazil; ^3^Laboratory of Medical Mycology – LIM-53, Hospital das Clínicas FMUSP and Instituto de Medicina Tropical de São Paulo, Universidade de São PauloSão Paulo, Brazil; ^4^Bruker do BrasilAtibaia, Brazil; ^5^bioMérieuxSão Paulo, Brazil

**Keywords:** MALDI-TOF mass spectrometry, *Candida haemulonii* complex species, Microflex LT^TM^, Vitek MS^TM^, ClinProTools^TM^

## Abstract

*Candida haemulonii* is now considered a complex of two species and one variety: *C. haemulonii* sensu stricto, *Candida duobushaemulonii* and the variety *C. haemulonii* var. *vulnera*. Identification (ID) of these species is relevant for epidemiological purposes and for therapeutic management, but the different phenotypic commercial systems are unable to provide correct species ID for these emergent pathogens. Hence, we evaluated the MALDI-TOF MS performance for the ID of *C. haemulonii* species, analyzing isolates/strains of *C. haemulonii* complex species, *Candida pseudohaemulonii* and *Candida auris* by two commercial platforms, their databases and softwares. To differentiate *C. haemulonii* sensu sctricto from the variety *vulnera*, we used the ClinProTools^TM^ models and a single-peak analysis with the software FlexAnalysis^TM^. The Biotyper^TM^ database gave 100% correct species ID for *C. haemulonii* sensu stricto, *C. pseudohaemulonii* and *C. auris*, with 69% of correct species ID for *C. duobushaemulonii*. Vitek MS^TM^ IVD database gave 100% correct species ID for *C. haemulonii* sensu stricto, misidentifying all *C. duobushaemulonii* and *C. pseudohaemulonii* as *C. haemulonii*, being unable to identify *C. auris*. The Vitek MS^TM^ RUO database needed to be upgraded with in-house SuperSpectra to discriminate *C. haemulonii* sensu stricto, C. *duobushaemulonii*, *C. pseudohaemulonii*, and *C. auris* strains/isolates. The generic algorithm model from ClinProTools^TM^ software showed recognition capability of 100% and cross validation of 98.02% for the discrimination of *C. haemulonii* sensu stricto from the variety *vulnera.* Single-peak analysis showed that the peaks 5670, 6878, or 13750 m/z can distinguish *C. haemulonii* sensu stricto from the variety *vulnera.*

## Introduction

The taxonomy of the pathogenic *Candida* species such as *Candida albicans*, *Candida parapsilosis*, and *Candida glabrata* has suffered significant modifications due to the description of closely related species (Turner and Butler, 2014). Likewise, the taxonomy of *Candida haemulonii* has changed over the years. In the early 90s, Lehmann et al. (1993), studying strains of *C. haemulonii* from distinct geographic origins and clinical sources, divided this species into two genetically distinct groups, named group I and II. This concept that *C. haemulonii* was indeed a complex of different species, was later confirmed by Cendejas-Bueno et al. (2012) that proposed a new classification: *C. haemulonii* (former group I), *Candida duobushaemulonii* (former group II) and the new variety, *C. haemulonii* var. *vulnera*. The correct identification (ID) of the *C. haemulonii* complex species is clinically relevant, since resistance to azole derivatives are commonly reported among the isolates of this species complex and amphotericin B has poor *in vitro* activity against *C. duobus haemulonii* isolates (Cendejas-Bueno et al., 2012; Ramos et al., 2015).

This increasing number of *Candida* species causing human infections has created a challenge for clinical laboratories to provide reliable and fast ID, especially for closely related species (Merseguel et al., 2015). Recently, the widespread commercial system Vitek 2^TM^ (bioMérieux, Marcy-L’Etoile, France) was linked to mis-IDs of *Candida auris* as *C. haemulonii* (Kathuria et al., 2015). Alternatively, reliable *Candida* species ID can be achieved by the sequence analysis of the internal transcribed spacer (ITS) region from the ribosomal DNA (rDNA; Schoch et al., 2012). However, the whole process of molecular analysis remains time consuming and costly. Thus, matrix-assisted laser desorption ionization-time of flight mass spectrometry (MALDI-TOF MS) emerges as a fast and accurate method for yeast’s ID in clinical microbiology laboratories (Bader, 2013; Jamal et al., 2014). This method produces species-specific protein fingerprints that can be compared with databases that contain reference or main mass spectra (MSP) of a great diversity of microorganisms, including yeasts (Bader, 2013). Conversely, the performance of different MALDI-TOF MS instruments in the ID of yeasts was found to vary considerably when compared to the conventional techniques as these systems often lack MSPs of cryptic *Candida* species in their databases (Jamal et al., 2014).

Initial evaluation using MALDI-TOF MS for the ID of *C. haemulonii* complex species provided promising results (Cendejas-Bueno et al., 2012), however, the discrimination of the variety *vulnera* from *C. haemulonii* sensu stricto was troublesome and the performance of the Vitek MS^TM^ (bioMérieux) remains unevaluated. Hence, aiming to provide a more detailed evaluation of the MALDI-TOF MS performance for the ID of *C. haemulonii* species ID, we analyzed a set of different isolates and reference strains belonging to the *C. haemulonii* complex species with the two available platforms, their databases and softwares.

## Material And Methods

### Isolates and Strains

**FIGURE 1:**
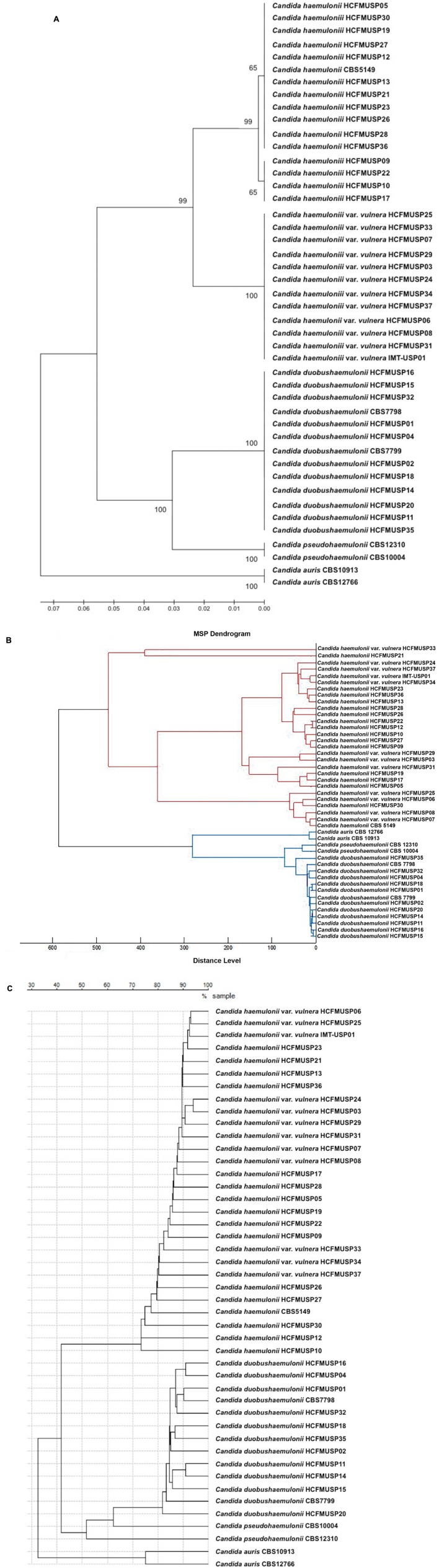
**Comparison of phylogenetic tree and MALDI-TOF dendrograms of the isolates and strains belonging to *Candida haemulonii* complex and close related taxa: **(A)** Phylogenetic tree obtained by UPGMA analyses and 1,000 bootstrap simulations based on ITS sequences; **(B)** Bruker Biotyper^TM^ dendrogram clustering of the MSPs with distances displayed in relative units on the *x* axis; **(C)** SARAMIS^TM^ dendrogram clustering of representative spectra from each isolate/strain with distances displayed in % similarities on the *x* axis**.

A total of 38 non-replicate clinical isolates belonging to the *C. haemulonii* complex species (15 *C. haemulonii*, sensu stricto, 12 *C. haemulonii* var vulnera, 11 *C. duobushaemulonii*) were analyzed in this study (**Table 1**). Species ID was carried out by ITS1 region sequence analysis with primers ITS1 and ITS4 and amplification parameters previously described (Luo and Mitchell, 2002). Both strands of purified ITS1 fragments were sequenced using the BigDye Terminator v.3.1 cycle sequencing kit on an ABI 3730 DNA analyzer (ABI, Foster City, EUA). Consensus sequence assembly and editing were performed using the software CondonCode Aligner, version 4.0 (CodonCode Corporation, Centerville, MA, USA), before being deposited in the GenBank database. In addition, a set of reference strains from the CBS-KNAW collection was also analyzed: *C. haemulonii* CBS5149^T^, *C. duobushaemulonii* CBS7798^T^, *C. duobushaemulonii* CBS7799. For specificity control, the reference strains belonging to the close related species *Candida pseudohaemulonii* (CBS10004 and CBS12370) and *C. auris* (CBS12766 and CBS10913) were also analyzed (**Table 1**). Phylogenetic analyses using UPGMA with 1,000 bootstrap simulations (**Figure 1A**) were conducted with Mega software, version 6.0 (Tamura et al., 2013).

**Table 1:** Denominations and GenBank accession numbers of the isolates and strains analyzed in this study.

Isolate/strain	GenBank acession no.	Species
HCFMUSP01	KT236448.1	*Candida duobushaemulonii*
HCFMUSP02	KT968725.1	*C. duobushaemulonii*
HCFMUSP03	KT236449.1	*Candida haemulonii* var. *vulnera*
HCFMUSP04	KT968726.1	*C. duobushaemulonii*
HCFMUSP05	KT968713.1	*Candida haemulonii*
HCFMUSP06	KT968733.1	*C. haemulonii* var. *vulnera*
HCFMUSP07	KT968734.1	*C. haemulonii* var. *vulnera*
HCFMUSP08	KT968735.1	*C. haemulonii* var. *vulnera*
HCFMUSP09	KT968714.1	*C. haemulonii*
HCFMUSP10	KT257659.1	*C. haemulonii*
HCFMUSP11	KT968727.1	*C. duobushaemulonii*
HCFMUSP12	KT968715.1	*C. haemulonii*
HCFMUSP13	KT968716.1	*C. haemulonii*
HCFMUSP14	KT968728.1	*C. duobushaemulonii*
HCFMUSP15	KT968729.1	*C. duobushaemulonii*
HCFMUSP16	KT968730.1	*C. duobushaemulonii*
HCFMUSP17	KT968717.1	*C. haemulonii*
HCFMUSP18	KT968731.1	*C. duobushaemulonii*
HCFMUSP19	KT968718.1	*C. haemulonii*
HCFMUSP20	KT968732.1	*C. duobushaemulonii*
HCFMUSP21	KT968719.1	*C. haemulonii*
HCFMUSP22	KT968720.1	*C. haemulonii*
HCFMUSP23	KT968721.1	*C. haemulonii*
HCFMUSP24	KT968736.1	*C. haemulonii* var. *vulnera*
HCFMUSP25	KT968737.1	*C. haemulonii* var. *vulnera*
HCFMUSP26	KT968722.1	*C. haemulonii*
HCFMUSP27	KT257660.1	*C. haemulonii*
HCFMUSP28	KT968723.1	*C. haemulonii*
HCFMUSP29	KT968738.1	*C. haemulonii* var. *vulnera*
HCFMUSP30	KT968724.1	*C. haemulonii*
HCFMUSP31	KT968739.1	*C. haemulonii* var. *vulnera*
HCFMUSP32	KU365080.1	*C. duobushaemulonii*
HCFMUSP33	KU365081.1	*C. haemulonii* var. *vulnera*
IMT-USP01	KX118598.1	*C. haemulonii* var. *vulnera*
HCFMUSP34	KX118599.1	*C. haemulonii* var. *vulnera*
HCFMUSP35	KX118600.1	*C. duobushaemulonii*
HCFMUSP36	KX118601.1	*C. haemulonii*
HCFMUSP37	KX118602.1	*C. haemulonii* var. *vulnera*
CBS5149^T^	AB118789.1	*C. haemulonii*
CBS7798^T^	JX459765.1	*C. duobushaemulonii*
CBS7799	JX459766.1	*C. duobushaemulonii*
CBS10004	AB118791.1	*Candida pseudohaemulonii*
CBS12310	JX459678.1	*C. pseudohaemulonii*
CBS10913	KF689018.1	*Candida auris*
CBS12766	KC692039.1	*C. auris*


### MALDI-TOF MS: Sample Preparation for Analysis

The isolates and strains were cultured on Sabouraud’s dextrose agar (SDA) plates and incubated for 48 h at 30°C before MALDI-TOF MS analysis. One loop of yeast biomass was transferred to a microtube containing 300 μl of purified water and final protein extraction protocol with absolute ethanol (Merck, Darmstadt, Germany) and formic acid 70% (Sigma-Aldrich, St. Louis, MO, USA) was carried out according to the Bruker’s recommendations.

One microliter of the crude protein extract of each isolate/strain was spotted onto a target plate. After air-drying, each spot was overlaid with 1 μl of HCCA matrix solution (Sigma-Aldrich).

### Bruker Daltonics MALDI-TOF MS Analysis

Measurements were performed on a Microflex LT^TM^ (Bruker Daltonics, Bremen, Germany) instrument using the software FlexControl^TM^ version 3.4 (Bruker Daltonics). Bacterial test standard (BTS, Bruker Daltonics) was used for mass calibration^TM^ instrument parameter optimization. Spectra were acquired in linear positive mode within a mass range from 2000 to 20,000 m/z with the manufacturer’s suggested settings using automated collecting spectra mode. Then, the obtained spectra were analyzed by standard pattern-matching algorithm using the MALDI Biotyper^TM^ 3.1 software (Bruker Daltonics), which compared the raw spectra with the reference spectra of the Bruker library (database version 3.3.1, 5627 reference spectra) by using the default settings. ID criteria were used as recommended by the manufacturer: a score ≥ 2.000 indicated species level ID, a score between 1.700 and 1.999 indicated ID to the genus level and a score < 1.700 was interpreted as no ID. For MainSpectra (MSP) and dendrogram construction, flat-liners and bad quality spectra were removed and additional measurements were carried out to obtain 20 spectra from each isolate/strain. Spectra were then loaded into Biotyper^TM^ 3.1 software (Bruker Daltonics) for MSP creation and dendrogram clustering construction with the default settings (distance measure: correlation; linkage: average; score oriented).

### bioMérieux MALDI-TOF MS Analysis

Measurements were performed on a Vitek MS^TM^ instrument (bioMérieux) equipped with both IVD and RUO (SARAMIS^TM^) databases (bioMérieux). For the IVD analysis, spectra were obtained using the Vitek MS^TM^ automation control and Myla software (bioMérieux) with the manufacturer’s suggested settings. For each acquisition group, a standard (*Escherichia coli* ATCC 8739) was included to calibrate the instrument and validate the run. The spectra were analyzed by the Vitek MS^TM^ v3.2 IVD database (bioMérieux) that contains spectral profiles covering 3555 species. The software compares the spectrum obtained to the expected spectrum of each organism or organism group (e.g., bacteria or fungi) and high confidence level ID was considered when single species showed probability of ID ≥ 60%. For the RUO analysis, spectra were generated using the Launchpad v2.8 software and compared to the SARAMIS^TM^ v.4.12 database (bioMérieux) that contains 720 SuperSpectra (each one corresponding to a spectral fingerprint with 15–20 species-specific biomarkers) of different fungal species. Peak matches that yield identification results with confidence values exceeding 75% were considered significant. For SuperSpectra and dendrogram construction, spectra of all isolates/strains were imported into the SARAMIS Premium^TM^ software package (bioMérieux). Then, SuperSpectra were calculated using the SARAMIS^TM^ SuperSpectrum tool (bioMérieux) according to the manufacturer’s instructions, and the specificity of the potential biomarker masses was determined by comparison against the whole SARAMIS^TM^ spectral archive (bioMérieux). Dendrogram was created based on whole spectra, with a single-link clustering algorithm and a binary mass list with an error of 800 ppm.

### Differentiation of *Candida haemulonii* sensu sctricto from the Variety *vulnera*

#### ClinProTools^TM^ Models

The ClinProTools^TM^ (Bruker Daltonics) generates multiple mathematical algorithms to generate pattern recognition models for classification and prediction of different classes (e.g., *C. haemulonii* sensu stricto class 1, *C. haemulonii* var. *vulnera* class 2) from mass spectrometry based profiling data. Moreover, ClinProTools^TM^ provides a list of peaks sorted according to the statistical significance to differentiate between both classes (Ketterlinus et al., 2005). Thus, for recognition of mass spectra patterns and biomarkers between *C. haemulonii* sensu stricto and *C. haemulonii* var. *vulnera*, spectra peak analysis models with ClinProTools^TM^ software v.3.0 (Bruker Daltonics) were created from 320 mass spectra of the 16 *C. haemulonii* sensu stricto (10 high-quality mass spectra per isolate) and 12 *C. haemulonii* var. *vulnera* (≈14 high-quality mass spectra per isolate) isolates. Spectra were pretreated with a resolution of 800 ppm, a mass range of 2000–20000 Da, a top hat baseline subtraction with 10% minimal baseline width, enabling null spectra exclusion, and recalibration with 500 ppm maximal peak shift and 30% match calibrant peaks. ClinProTool^TM^ smodels (Bruker Daltonics) were generated using three algorithms: Genetic Algorithm (GA), Supervised Neural Network (SNN), and Quick Classifier (QC). For each model, the recognition capability (RC) and cross validation (CV) percentage were generated to demonstrate the reliability and accuracy of the model. RC and CV percentages were indicators of the model’s performance and useful predictors of the model’s ability to classify test isolates. The model with the highest RC and CV values were used in the analysis.

#### Single-Peak Analysis

For each peak, the AUC for the discrimination of the groups was directly obtained from the ClinProTools^TM^ v.3.0 software (Bruker Daltonics). For the five peaks with the highest AUC, the detection performances were checked using FlexAnalysis^TM^ v.3.4 (Bruker Daltonics). After smoothing and baseline subtraction, the mass lists for each isolate were obtained using the centroid algorithm with a signal-to-noise (SN) threshold of 0.5 and a maximum of 500 peaks and exported to Microsoft Excel. The SN ratios of the peaks with a tolerance of 1.000 ppm were exported to SPSS 18.0. ROC curves were constructed, and their optimal cutoff values were determined with the maximum Youden index.

## Results

### Bruker Biotyper^TM^

The Bruker Biotyper^TM^ (Bruker Daltonics) gave correct species with scores ≥ 2.0 for all *C. haemulonii* sensu stricto isolates/strains (**Table 2**). For the species *C. duobushaemulonii*, correct species ID (score ≥ 2.0) was achieved for seven isolates and the two CBS strains (69%), while four isolates were assigned as *C. duobushaemulonii* with a score between 1.797 and 1.935. These isolates were analyzed at least two times and the results were confirmed. After the inclusion in the Biotyper^TM^ (Bruker Daltonics) database of MSPs from two Brazilian isolates (HCFMUSP04 and HCFMUSP11), all mass spectra of that species had scores above 2.3. All reference strains of *C. pseudohaemulonii* and *C. auris* had correct species ID with scores ≥ 2.0. All *C. haemulonii* var. *vulnera* isolates were assigned as *C. haemulonii* sensu stricto with scores ≥ 2.0. The dendrogram generated by the Biotyper^TM^ (**Figure 1B**) show the clustering of MSPs of the isolates/strains of *C. haemulonii* sensu stricto and *C. haemulonii* var. *vulnera* in the same node, exemplifying the similarity of these MSPs.

**Table 2:** **Performance of the different databases for the identification of *C. haemulonii* sensu stricto, *C. haemulonii* variety *vulnera*, *C. duobushaemulonii*, *C. pseudohaemulonii*, and *C. auris* isolates/strains**.

**Species/variety**	**Bruker Microflex LT^TM^**		**VITEK MS^TM^**		
		
	**Biotyper^TM^ v.3.1 (% with LS^1^ ≥ 2)**	**Biotyper^TM^ v.3.1 with in-house MSPs^2^ (% with LS ≥ 2)**	**IVD (% with CI^3^ ≥ 60%)**	**RUO (% with CI ≥ 75%)**	**RUO with in-house SuperSpectra (% with CI ≥ 75%)**
*Candida haemulonii* sensu stricto (*n* = 16)	16/16 (100)	16/16 (100)	16/16 (100)	0/16 (0)	16/16 (100)
*C. haemulonii* var. *vulnera* (*n* = 12)	0/0 (0)	0/12 (0)^4^	0/12 (0)^4^	0/12 (0)	0/12 (0)^4,5^
*Candida duobushaemulonii* (*n* = 13)	9/13 (69)	13/13 (100)	0/13 (0)^4^	0/13 (0)	13/13 (100)
*Candida pseudohaemulonii* (*n* = 2)	2/2 (100)	2/2 (100)	0/2 (0)^4^	0/2 (0)	2/2 (100)
*Candida auris* (*n* = 2)	2/2 (100)	2/2 (100)	0/2 (0)	0/2 (0)	2/2 (100)


*^1^LS, logscore values; ^2^MSP, main spectrum profile; ^3^CI, confidence interval for a single species; ^4^misidentified as *C. haemulonii* sensu stricto; ^5^not able to find specific masses for the creation of SuperSpectra for the var. *vulnera.**

### Vitek MS^TM^ Species Identification

The bioMérieux Vitek MS^TM^ IVD gave correct species with with 99.9% level of ID for all *C. haemulonii* sensu stricto isolates/strains (**Table 2**). However, all *C. duobus haemulonii* isolates/strains and the two reference strains of *C. pseudohaemulonii* were misidentified as *C. haemulonii* with 99.9% level of ID in at least two separate experiments, while the two strains of *C. auris* had no species ID. The Vitek MS^TM^ RUO (SARAMIS^TM^) analysis gave neither genus nor species ID for all isolates and strains (**Table 2**). After the upgrade of the SARAMIS^TM^ database with SuperSpectra of *C. haemulonii*, C. *duobushaemulonii*, *C. pseudohaemulonii* and *C. auris*, all isolates/strains belonging to these species had correct species assignment (**Table 2**). It was not possible to create a SuperSpectrum of *C. haemulonii* var *vulnera* with at least 15 masses that would have differentiated it from *C. haemulonii* sensu stricto. The dendrogram generated by the SARAMIS^TM^ (bioMérieux) software showed similar clustering results to the dendrogram generated by the Biotyper^TM^ software, gathering *C. duobushaemulonii, C. pseudohaemulonii, and C. auris* into three species-specific nodes. However, *C. haemulonii* sensu stricto and the *variety vulnera* were found mixed inside the same node (**Figures 1B,C**).

### Differentiation of *Candida haemulonii* sensu sctricto from the Variety *vulnera*

**FIGURE 2:**
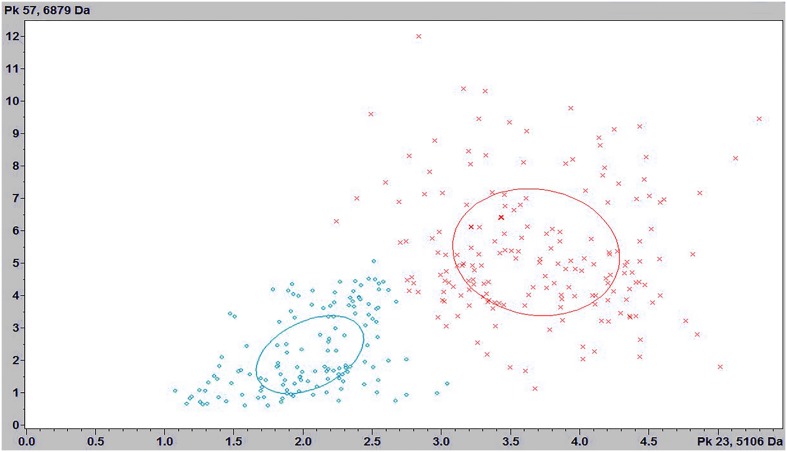
Strain distribution map corresponding to *C. haemulonii* sensu stricto (green) and *C. haemulonii* var. *vulnera* (red).

**FIGURE 3:**
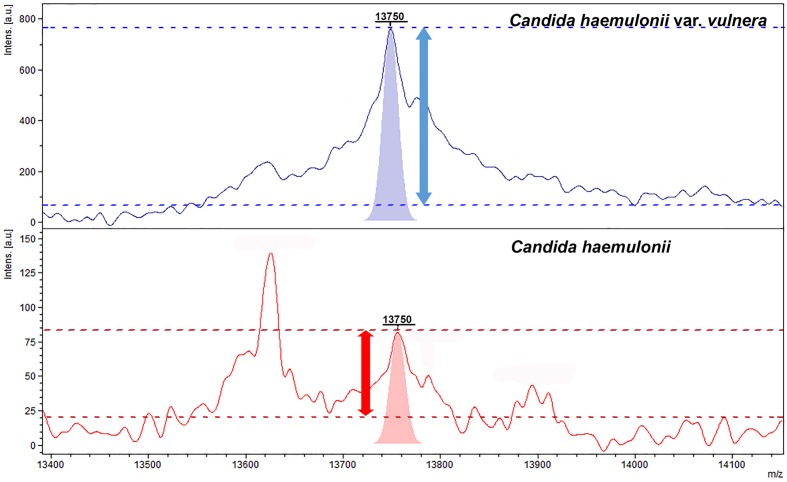
**Representative mass spectra of *C. haemulonii* sensu stricto (red) and *C. haemulonii* var. *vulnera* (purple) between 13400 and 14100 Da showing the peak intensities and peak areas of the 13750 Da peak.** Horizontal dashed lines depict the signal (above) and noise (below) intensity values used to calculate the signal-to-noise ratios.

The three classification models from ClinProTools^TM^ showed values of RC above ≥90% for the discrimination of *C. haemulonii* sensu stricto and *C. haemulonii* var *vulnera*. The best results were provided by the GA model, with RC and CV of 100 and 98.02%, respectively. The strain distribution maps based on the GA clearly show that *C. haemulonii* sensu stricto and the variety *vulnera* can be divided into one of two categories based on their peptide mass fingerprints (**Figure 2**). The peaks that had the highest AUC (>0.9) for the discrimination of the *C. haemulonii* sensu strito and *C. haemulonii* var. *vulnera* by ClinProTools^TM^ were 5106, 5670, 6878, 13750, and 14046 m/z. However, the performances of these peaks for the discrimination of the two groups using the FlexAnalysis^TM^ software showed that only the peaks 5670, 6878, and 13750 m/z had AUC > 0.9, with sensitivity and specificity of 94.7, 92.4, 94.1%, and 77.0, 94.5, 96.1%, respectively. The SN cut-off values of the peaks 5670, 6878, and 13750 m/z for the discrimination of the *C. haemulonii* (below cut-off) and *C. haemulonii* var. *vulnera* (above cut-off) were 3.1, 2.86, and 6.04, respectively. The ClinProTools^TM^ and single-peak analysis results for the differentiation of *C. haemulonii* sensu stricto from the variety *vulnera* are summarized in **Table 3** and exemplified in **Figure 3**.

**Table 3:** Single-peak analysis for the discrimination of *C. haemulonii* sensu stricto and *C. haemulonii* variety *vulnera.*

**Peak (m/z)**	**ClinProTools^TM^**				**FlexAnalysis^TM^**					
		
	**AUC^1^**	**Dave^2^**	**PWKW^3^**	**PAD^4^**	**Ave1^5^**	**Ave2**	**AUC^6^**	**Cut-off^6^**	**Sensitivity (%)**	**Specificity (%)**
5106	0.95	1.82	<0.0001	<0.0001	10.46	17.88	0.81	2.77	96.5	74.86
5670	0.93	1.94	<0.0001	<0.0001	16.65	18.85	0.90	3.01	94.77	77.05
6878	0.92	3.51	<0.0001	<0.0001	12.89	15.53	0.95	2.89	92.44	94.54
13750	0.96	1.51	<0.0001	<0.0001	5.09	59.01	0.98	6.04	94.19	96.17
14046	0.95	0.27	<0.0001	0.00335	23.37	26.06	0.77	2.01	70.35	77.60


*Peaks with the best performances according to ClinProTools^TM^ and FlexAnalysis^TM^ softwares.*

*^1^AUC, area under the curve; ^2^Dave, difference between the maximal and the minimal average peak area/ intensity of the groups; ^3^PWKW, *p*-value of Wilcoxon/Kruskal–Wallis test (range: 0–1; 0 = good); ^4^PAD: *p*-value of Anderson–Darling test, <0.05 indicates data not normally distributed; gives information about normal distribution (range: 0–1; 0 = not normal distributed); ^5^Ave, area/intensity average of a group from *C. haemulonii* sensu stricto (1) and *C. haemulonii* var. *vulnera* (2); ^6^AUCs and signal-to-noise cutoff values were obtained from ROC curve constructed by SPSS Version 18.0 with the using of FlexAnalysis.*

## Discussion

The taxonomy of the genus *Candida* has been suffered significant changes due to the description of new closely related species, with some species as *C. haemulonii* being considered as cryptic species complex (Brandt and Lockhart, 2012). The discrimination of these cryptic *Candida* species from their closest relatives is providing new information regarding their epidemiology, pathogenicity and clinical significance (Brandt and Lockhart, 2012; Cendejas-Bueno et al., 2012; Nobrega de Almeida et al., 2016).

In this context, MALDI-TOF MS has been successfully applied to discriminate cryptic species from the genus *Candida*, such as *C. parapsilosis, Candida orthopsilosis*, and *Candida metapsilosis* (Quiles-Melero et al., 2012; Nobrega de Almeida et al., 2014), *C. glabrata*, *Candida nivariensis*, and *Candida bracariensis* (Pinto et al., 2011), *C. albicans* and *Candida dubliniensis* (Pinto et al., 2011; Jamal et al., 2014), and more recently, it has been evaluated for the differentiation of the *C. haemulonii* species complex and other close related taxa (Cendejas-Bueno et al., 2012; Kathuria et al., 2015).

The Bruker’s Biotyper^TM^ v.3.1 is currently the most adapted database for the ID of *C. haemulonii* species, presenting with 08, 04, and 07 MSPs of *C. haemulonii, C. haemulonii* var. *vulnera* and *C. duobushaemulonii*, respectively. However, four *C. duobushaemulonii* isolates, despite showing the best match results with the MSPs of the same species, had ID values below 2.0. After the expansion of the database with in-house MSPs, all isolates had ID values above 2.0. This illustrates the need for the Bruker database expansion with local well-identified isolates to reach optimal results, as previously reported by other authors (Cassagne et al., 2011; de Almeida Júnior et al., 2014).

The IVD database from the Vitek MS^TM^ includes only the species *C. haemulonii*. Surprisingly, the different taxa *C. duobushaemulonii* and *C. pseudohaemulonii* were misidentified as *C. haemulonii*. Since these species have well-distinct spectral profiles, the misidentifications may be related to the inclusion of strains without updated species ID when this database was constructed. The update of the Vitek MS^TM^ IVD database will certainly enhance its ability to correctly ascertain the different species from *C. haemulonii* complex and the close related taxa, since this system has proven to well-differentiate phylogenetically similar microorganisms, such as *Streptococcus pneumoniae* from species of the *Streptococcus mitis* group (Branda et al., 2013). The RUO database (SARAMIS^TM^) has proven to be an auxiliary tool when the IVD database fails to provide correct yeast species ID (Chao et al., 2014). In the case of *C. haemulonii* complex species, the inclusion of SuperSpectra was necessary to optimize the performance of the Vitek MS^TM^. Nevertheless, we demonstrated the specificity of the SARAMIS database, since no mis-IDs were reported, which was not the case of the IVD database.

The ClinProTools^TM^ software is a biomarker analyzer that has been widely applied in bacteriology, providing rapid and cost-saving method for epidemiological clustering, strain typing (Xiao et al., 2014; Angeletti et al., 2015; Zhang et al., 2015), and for detection of staphylococcal Panton-Valentine leukocidin (Bittar et al., 2009). This software generates classification models from large numbers of spectra and detects small differences among different clusters, based on mass, signal-to-noise, intensity, peak heights and peak areas. Moreover, allying ClinProTools^TM^ and single-peak analysis with FlexAnalysis^TM^ has proven to provide higher discriminatory power to detect bio-marker peaks (Angeletti et al., 2015; Zhang et al., 2015). However, ClinProtools^TM^ was until now an unexplored tool for the differentiation of close taxa in the fungi kingdom.

In conclusion, we show that the Biotyper^TM^ database 3.1 performs relatively well for discriminating *C. haemulonii* species and close related taxa, but requires addition of MPSs representing the local diversity to achieve optimal results, while Vitek MS^TM^ databases perform less well and need major update. Moreover, we describe here the most discriminatory peaks along with the SN cut-off values that can differentiate *C. haemulonii* and the variety *vulnera* with a simple inspection of the mass spectra profile in routine basis software such as FlexAnalysis^TM^.

## Author Contributions

JA: design the study, helped with acquisition and data analysis, drafted and revised the work, approved the final work and agrees with all the aspects of the work; RG, AS, GD: helped with acquisition and data analysis, revised the work, approved the final work, and agree with all the aspects of the work; VG, RM, DA, AR, AM: helped with acquisition of the data, revised the work, approved the final work and agree with all the aspects of the work; FR, LJ: helped with data analysis, revised the work, approved the final work and agree with all the aspects of the work; GB: helped with data analysis, drafted and revised the work, approved the final work and agrees with all the aspects of the work.

## Conflict of Interest Statement

The authors declare that the research was conducted in the absence of any commercial or financial relationships that could be construed as a potential conflict of interest.
